# Supplementary material for: Brazilian Clinical Strains of Actinobacillus pleuropneumoniae and Pasteurella multocida: Capsular Diversity, Antimicrobial Susceptibility (In Vitro) and Proof of Concept for Prevention of Natural Colonization by Multi-Doses Protocol of Tildipirosin
Source: Antibiotics (Basel). 2023 Nov 25;12(12):1658. doi: 10.3390/antibiotics12121658 (PMC10740920; doi:10.3390/antibiotics12121658)
Supplement: Supplementary file 1 [file antibiotics-12-01658-s001.zip › antibiotics-2631320-supplementary.pdf]

Supplementary material

Table S1. Results of the *p* values obtained for the statistical analysis performed for the *A. pleuropneumoniae* tildipirosin susceptibility percentages and the variables "Central or Southern Brazilian regions", "isolation year", "origin from farm or slauterhouses" and "*A. pleuropneumoniae* serovar".

Variables compared	Rate of susceptibility (%)	two-tailed *p* value
Tildipirosin versus:		
Central states	97.1	0.171
Southern states	90.3	
2014–2015	100.0	0.185
2016–2017	90.7	
2018	96.2	
Farms	100.0	0.068
Slaughterhouses	91.2	
Serovar 1	100.0	0.075
Serovar 5	100.0	
Serovar 6	100.0	
Serovar 7	92.0	
Serovar 8	95.3	
Serovar 14	100.0	
Non-typable isolates	100.0	

Table S2. Results of the *p* values obtained for the statistical analysis performed for *P. multocida* tildipirosin susceptibility percentages and the variables "Central or Southern Brazilian regions", "isolation place", "pig age", and "presence of absence of *P. multocida pfhA* gene.

Variables compared	Rate of susceptibility (%)	two-tailed *p* value
Tildipirosin versus:		
Central states	75.0	0.287
Southern states	50.0	
2017	70.0	0.379
2018	72.7	
2019	70.4	
2020	100.0	
2021	50.0	
Farms	71.9	1.000
Slaughterhouses	75.0	
≤ 30 days	33.3	0.223
31–70 days	66.7	
> 70 days	77.1	
pfhA +	60.0	0.433
pfhA -	76.0	
